# Discovering and Targeting Dynamic Drugging Pockets of Oncogenic Proteins: The Role of Magnesium in Conformational Changes of the G12D Mutated Kirsten Rat Sarcoma-Guanosine Diphosphate Complex

**DOI:** 10.3390/ijms232213865

**Published:** 2022-11-10

**Authors:** Zheyao Hu, Jordi Marti

**Affiliations:** Department of Physics, Polytechnic University of Catalonia-Barcelona Tech, B5-209 Northern Campus UPC, 08034 Barcelona, Catalonia, Spain

**Keywords:** benzothiadiazine derivatives, drug design, molecular dynamics, KRAS oncogenes

## Abstract

KRAS-G12D mutations are the one of most frequent oncogenic drivers in human cancers. Unfortunately, no therapeutic agent directly targeting KRAS-G12D has been clinically approved yet, with such mutated species remaining undrugged. Notably, cofactor Mg${}^{2+}$ is closely related to the function of small GTPases, but no investigation has been conducted yet on Mg${}^{2+}$ when associated with KRAS. Herein, through microsecond scale molecular dynamics simulations, we found that Mg${}^{2+}$ plays a crucial role in the conformational changes of the KRAS-GDP complex. We located two brand new druggable dynamic pockets exclusive to KRAS-G12D. Using the structural characteristics of these two dynamic pockets, we designed in silico the inhibitor DBD15-21-22, which can specifically and tightly target the KRAS-G12D-GDP-Mg${}^{2+}$ ternary complex. Overall, we provide two brand new druggable pockets located on KRAS-G12D and suitable strategies for its inhibition.

## 1. Introduction

KRAS proteins play an important role in various cellular processes, such as cell proliferation, differentiation, and survival [1,2]. As a critical hub in cell signaling networks, KRAS functions as a binary molecular switch [3]: it can be activated by upstream receptors such as EGFR [4,5,6] and can regulate the downstream MAPK [7,8], PI3K [9,10] and RALGDS–RAL [11,12] pathways. KRAS mutations impair both the intrinsic and GAP stimulated GTP hydrolysis activity [13], leading to the hyperactivation of KRAS signaling and ultimately cancer. Plenty of studies have been devoted to the study of the different relevant mutations (G12, G13, Q61 among others) and to the interplay between GDP and GTP [14,15,16,17,18]. After near 40 years of efforts, a major breakthrough was made for KRAS-G12C inhibition [19]. The covalent binding of designed inhibitors to CYS12 induces a targetable allosteric switch-II pocket, eventually blocking KRAS-G12C [20,21,22]. Later on, different new covalent inhibitors targeting KRAS-G12C, such as AMG510 [23,24] and MRTX849 [25], were reported. In 2021, *Sotorasib* was finally approved by the U.S. Food and Drug Administration for the clinical treatment of KRAS-G12C mutant cancers [26,27].

In particular, several strategies have been proposed for targeting KRAS-G12D, such as indole-based inhibitors (BI-2852) of the Switch-I/II pockets [28,29], the piperazine-based compound TH-Z835 for ASP12 [30], the KRAS allosteric ligand KAL-21404358 targeting the site PRO110 [31], a multivalent small molecule named 3144 for pan-RAS inhibition [32] and a three cyclic peptide for GTP-bound KRAS-G12D [33]. It has only been in recent years that more works about G12D, G12R, G12S and G12V were reported and some specific inhibitors were proposed [34,35,36,37,38,39]. Despite these progresses, targeting the most prevalent and oncogenic KRAS-G12D mutation has remained elusive; no therapeutic agent has been clinically approved yet. Therefore, how to interrupt mutated KRAS-G12D oncogene remains a real challenge both in clinical and scientific research.

Clinical data have implicated not only that mutated RAS isoforms vary by tissue and cancer types, but also within the most frequently KRAS-mutations, the mutated amino acid residues of KRAS are also different for each cancer. For instance, among KRAS mutation predominant cancers, KRAS-G12C mutations mainly occur in lung cancer (45%), whereas KRAS-G12D mutations are related to more than 50% of pancreatic ductal adenocarcinoma cases [40,41,42]. The KRAS mutations display high tissue-specific abilities to drive tumorigenesis, strongly suggesting that the mutation of only one KRAS residue can lead to significant mechanistic changes in KRAS behavior. This usually leads to consider that there are only minor differences at the molecular scale between different KRAS mutants, such as one-site GLY12 substitutions. Therefore, the detailed information on atomic interactions and local structures at the all-atom level of KRAS can be of crucial importance for oncogenic KRAS research [43]. Until now, several studies have been published on the effects of G12D mutation on the structure and conformation changes of KRAS [39,44,45,46,47,48]. However, previous studies mainly focus on oncogenic proteins themselves and are less associated with the KRAS surroundings, although cancer is closely related to oncoproteins and the surrounding environment [49,50]. So, more detailed conformations and local structures of KRAS still remain to be revealed.

As one of the important RAS cofactors, the ion Mg${}^{2+}$ is coordinated in an octahedral arrangement with high affinity on RAS proteins [51,52,53]. Mg${}^{2+}$ has been established essential for both guanine nucleotide binding and GTP-hydrolysis of RAS proteins. For instance, in HRAS, the difference in affinity between HRAS and guanine nucleotide in the presence or absence of Mg${}^{2+}$ is ∼500-fold [54,55,56]. However, HRAS is rarely mutated in human cancers with only ∼10% rate found in bladder and cervical cancers [40,41,42]. The mechanism of Mg${}^{2+}$ interaction with the most prevalent oncogenic KRAS species has not been investigated yet. In this paper, using microsecond scale molecular dynamics (MD) simulations at an all-atom level, we reveal that cofactor Mg${}^{2+}$ plays a crucial role in the conformational changes of KRAS. The mutation of GLY12 site in KRAS-G12D triggers a distinct shift in the interaction patterns between Mg${}^{2+}$ and KRAS, finally generating two druggable exclusive dynamic pockets on the KRAS-G12D-GDP-Mg${}^{2+}$ ternary complex surface. In this paper, different from other works in the literature [46], we do not consider the association with the GTP species (which makes KRAS active) since it is related to the signaling of the oncogene, which can occur when attached to the cell membrane surface. Conversely, we consider the KRAS-GDP association (i.e., in the inactive state), which does not require the hugely computationally expensive incorporation of the cell membrane in an explicit way.

With full use of the structural characteristics of these two dynamic pockets we designed in silico the specific inhibitor DBD15-21-22, a derivative of benzothiadiazine [57,58] (DBD), which can specifically and tightly target the KRAS-G12D oncogene, stabilizing the inactive state of KRAS-G12D-GDP. The reliability of this finding was validated by subsequent molecular dynamics simulations of KRAS-G12D together with DBD15-21-22, where, by analyzing the differences between the three-dimensional structure of DBD15-21-22 and guanine nucleotide and combining them with the molecular dynamics simulation results of DBD15-21-22 and wild-type KRAS, we can propose that DBD15-21-22 will be harmless for wild-type KRAS.

## 2. Results and Discussion

**Figure A1:**
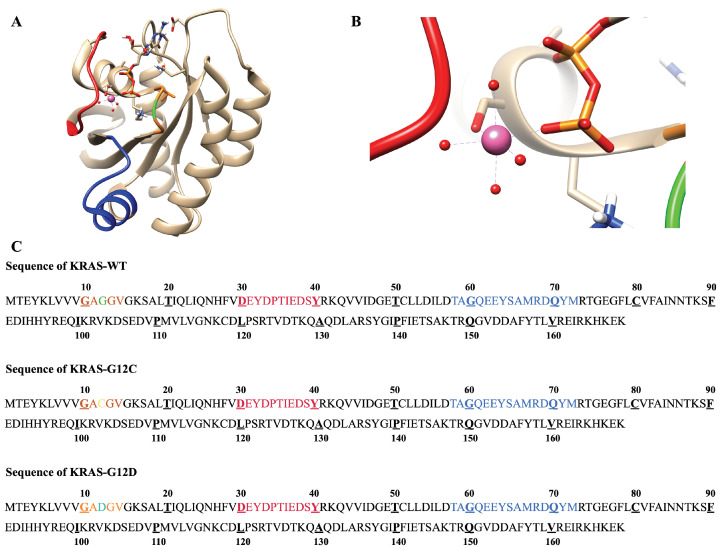
Initial structure and sequence of three KRAS isoforms: (**A**) SW-I (red), SW-II (blue), mutation site GLY12 (green); (**B**) Details of the octahedral structure of Mg${}^{2+}$ in the crystal structure; (**C**) Sequence of KRAS-WT, KRAS-G12C and KRAS-G12D. G12 (green), C12 (yellow), D12 (cyan).

**Figure A2:**
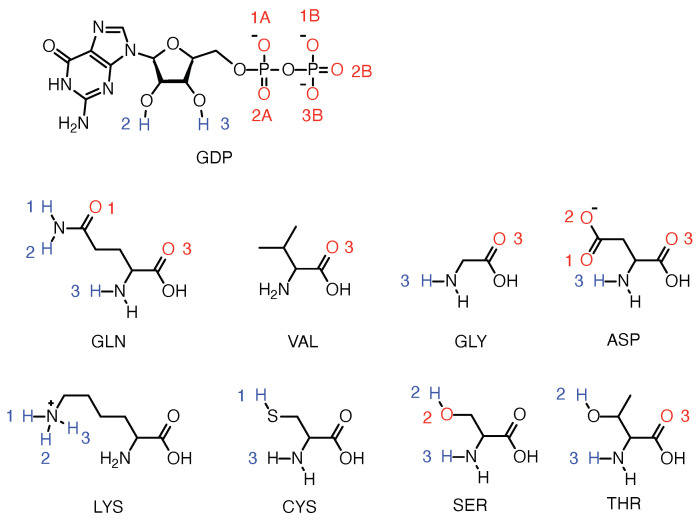
Sketches of GDP, and amino acid residues of KRAS protein mentioned in the text.

Firstly, we investigated the conformational changes of the three different isoforms of KRAS in aqueous ionic solution. The only difference between these three KRAS is located at codons 12, with the sequences and initial structures of the three isoforms shown in Figure A1 (Appendix A). Atomic detailed sketches of GDP and the main residues described in this part are reported in Figure A2. All meaningful atom–atom distances as a function of time and bond lifetimes are reported in Appendix A.

### 2.1. The Fluctuations and Stability of KRAS Protein Conformations with Different Mutation Isoforms

**Figure A3:**
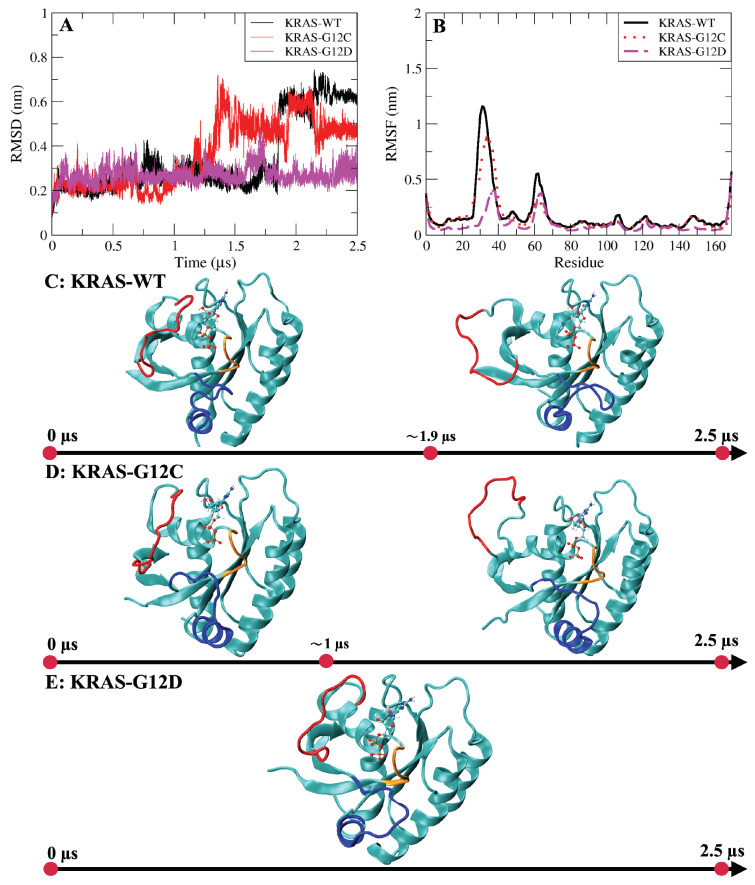
(**A**) Comparison of RMSD values of three KRAS proteins; (**B**) comparison of RMSF values of three KRAS proteins; (**C**) the evolution of KRAS-WT conformational changes; (**D**) the evolution of KRAS-G12C conformational changes; (**E**) the evolution of KRAS-G12D conformational changes. Red: Switch-I; blue: Switch-II; orange: P-loop. Water and ions have been hidden.

Root mean square deviations and root mean square fluctuations of KRAS-WT and the two mutant isoforms were firstly analyzed (Figure A3A,B). Both properties are defined below. RMSD results show the fluctuations and stability of the conformations of the three species. An overall view of the evolution of conformational changes is shown in Figure A3C–E. We found that for KRAS-WT, there was a distinct conformational fluctuation around 1.9 μs during the simulation. In a similar fashion, for KRAS-G12C, a large conformation change was observed after 1 μs. KRAS-G12D exhibited a distinct conformational stability different from that of KRAS-WT and KRAS-G12C, with overall stability and no significant changes. From the perspective of residues, RMSF reveals its flexibility during the full simulation. Switch-I (SW-I) and Switch-II (SW-II) are the regions of the protein which were recently identified as potential drugging sites [42,48,59], and they show high conformational flexibility compared to other structures of KRAS. Noticeably, we observed that the flexibility of residues in the SW-I domain of KRAS-G12D is around 2-fold smaller than that of KRAS-WT and KRAS-G12C. Combining the RMSD and RMSF results, the KRAS-G12D demonstrated significant stability along full statistics 5 μs MD simulations (2.5 μs per trajectory). This suggests that (1) the different isoforms of KRAS are not sharing the same mechanisms in conformational changes; and (2) it corroborates that to a large extent, the conformational change of the KRAS protein is mainly embodied by SW-I and SW-II. These findings are in overall good qualitative agreement with previous computational studies [60].

### 2.2. Mutation of GLY12 Enhances the Interaction between P-Loop and SW-II Domain

**Figure A4:**
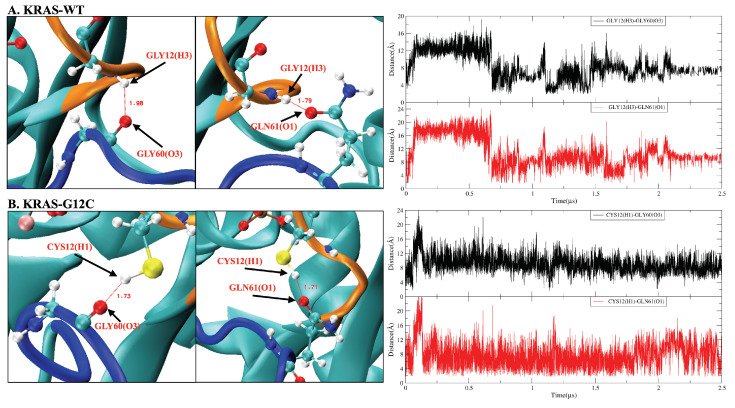
Hydrogen bond interactions between KRAS mutation site 12 and amino acid residues (GLY60, GLN61) of SW-II domain. (**A**) KRAS-WT case. Left side: representative snapshot of $HB_{GLY12(H3)-GLY60(O3)}$ and $HB_{GLY12(H3)-GLN61(O1)}$, right side: time evolution of atom–atom distance d(t) corresponding to the left side; (**B**) KRAS-G12C case. Left side: representative snapshot of $HB_{CYS12(H1)-GLY60(O3)}$ and $HB_{CYS12(H1)-GLN61(O1)}$, right side: time evolution of atom–atom distance d(t) corresponding to the left side.

**Figure A5:**
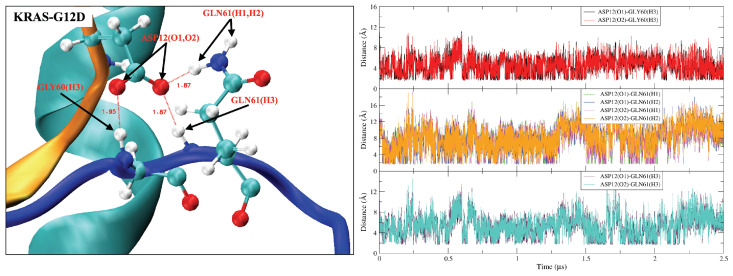
Hydrogen bond interactions between KRAS-G12D ASP12 residue and amino acid residues (GLY60, GLN61) of SW-II domain. (**Left**) Representative snapshot of $HB_{ASP12(O1/O2)-GLY60(H3)}$, $HB_{ASP12(O1/O2)-GLN61(H1/H2)}$ and $HB_{ASP12(O1/O2)-GLN61(H3)}$; (**Right**) time evolution of atom–atom distance d(t) corresponding to the (**Left**) figure.

**Figure A6:**
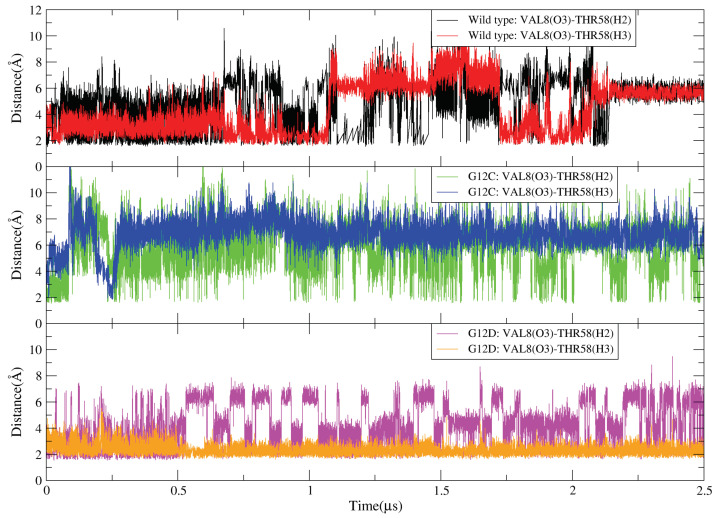
Atom-atom distance between oxygen atom (O3) of residue VAL8 and hydrogen atom (H2 and H3 of THR58) of KRAS SW-II.

The GLY12 mutation of KRAS protein also shows significant effects on the interaction of P-loop with Switch-II (Figure A4 and Figure A5). GLY12 has weak hydrogen-bonding (HB) interactions with GLY60 and GLN61, but such HB interactions are enhanced when the GLY12 mutated to CYS or ASP. Compared with the GLY12 of KRAS-WT, residue CYS12 contains a sulfhydryl group such that the HB interaction between CYS12 (H1) and GLN61 (O1) is mildly enhanced (see Figure A4B). When GLY12 mutates to ASP, it contains two active HB electron donor sites (carboxyl group). This reverses the surface electrical properties of residue 12, with ASP12 having a longer side chain. The interaction between position 12 and residues GLY60 and GLN61 is significantly enhanced so that they can form three simultaneous strong HB with GLY60 and GLN61 (Figure A5). In addition, the mutation of GLY12 also affects the interaction of VAL8 (near the P-loop) with THR58 (located on SW-II) (Figure A6). In the KRAS-WT case, the $HB_{VAL8(O3)-THR58(H2)}$ and $HB_{VAL8(O3)-THR58(H3)}$ are alternatively generated, but the lifetime of $HB_{VAL8(O3)-THR58(H2)}$ is slightly longer than that of $HB_{VAL8(O3)-THR58(H3)}$. In the KRAS-G12C case, the lifetime of $HB_{VAL8(O3)-THR58(H2)}$ is increased so that such a pair remains present for the entire simulation period, while $HB_{VAL8(O3)-THR58(H3)}$ interactions are mostly eliminated. Furthermore, in the case of KRAS-G12D, the lifetimes of both $HB_{VAL8(O3)-THR58(H2)}$ and $HB_{VAL8(O3)-THR58(H3)}$ are significantly enhanced, with these two HB being present throughout the whole simulation time. Notably, $HB_{VAL8(O3)-THR58(H3)}$ was more stable than $HB_{VAL8(O3)-THR58(H2)}$ in the KRAS-G12D case, which is a clear difference compared to KRAS-WT and KRAS-G12C.

### 2.3. Strong Coordination Interactions between Mg${}^{2+}$ and SER17, ASP57, GDP in KRAS-G12D

**Figure 1:**
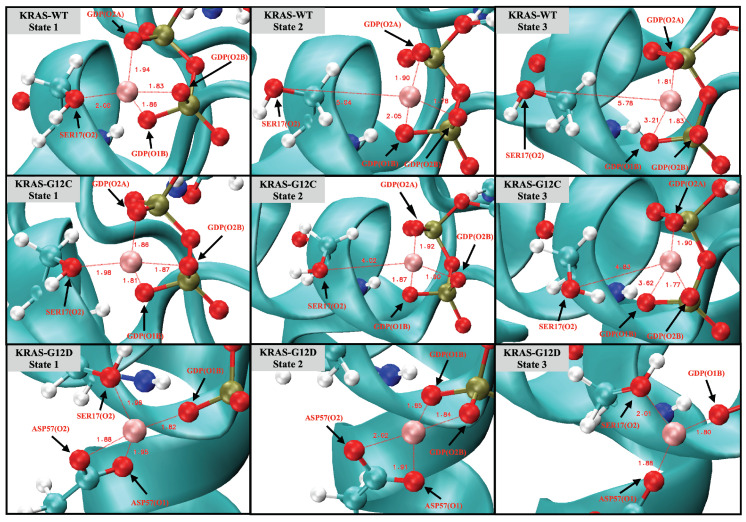
Snapshots of the coordination bonds between Mg${}^{2+}$ and SER17, ASP57 and GDP. Water molecules are hidden for the sake of clarity.

**Figure A7:**
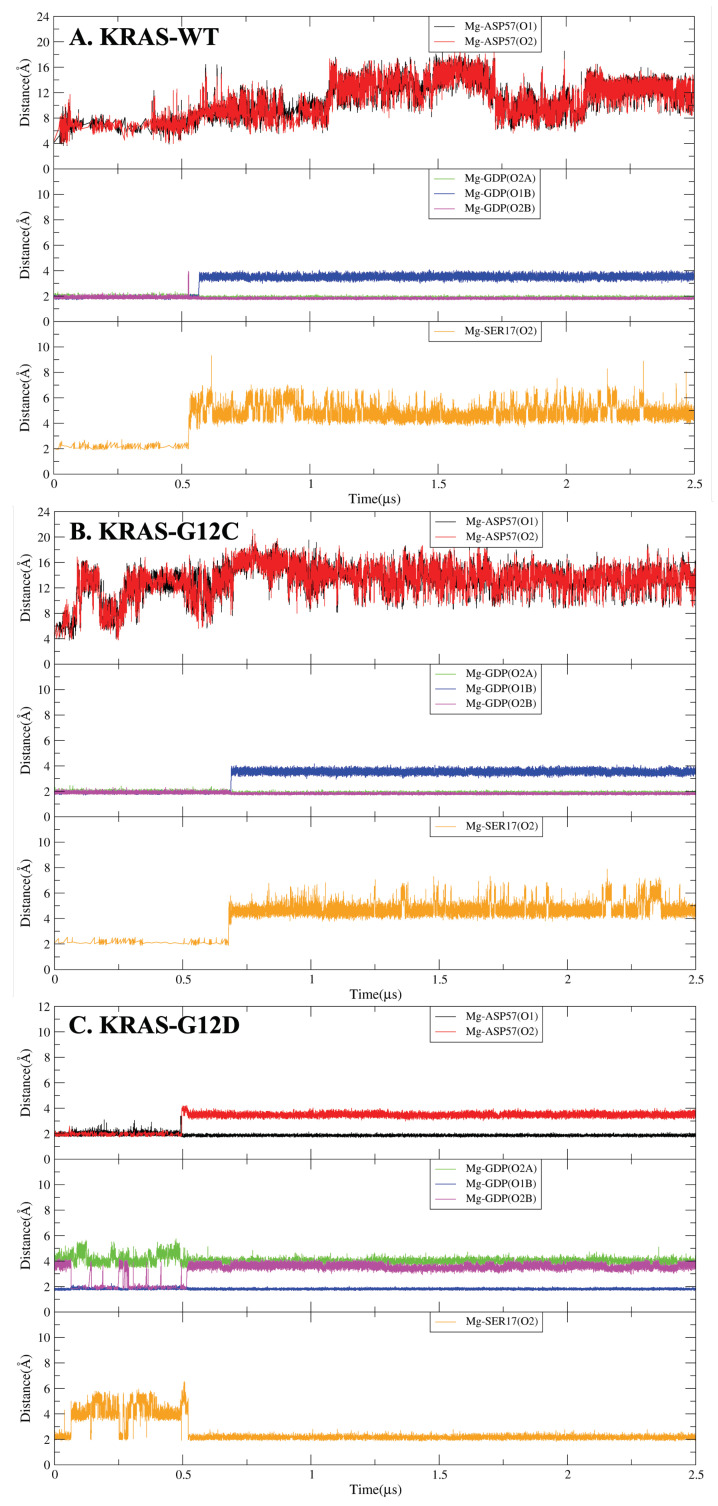
Coordination bonds analysis of Mg${}^{2+}$ with related amino acid residues and GDP. (**A**) The Mg${}^{2+}$ interaction with KRAS-WT-GDP; (**B**) the Mg${}^{2+}$ interaction with KRAS-G12C-GDP; (**C**) the Mg${}^{2+}$ interaction with KRAS-G12D-GDP.

Noncovalent interactions, such as hydrogen bonds and coordination bonds (CB), are important for proteins to maintain their tertiary structure as well as for protein–cofactor interactions. We analyzed the structure of the three different KRAS isoforms in the aqueous ionic solution by time-dependent atomic site–site distances between selected atomic sites to find out and estimate interactions between Mg${}^{2+}$ and KRAS (Figure A7). The full set of averaged values and lifetimes of HB/CB are also reported in Table A2 and Table A3 of Appendix A. We selected in Figure 1 representative snapshots from the total computed length of 5 μs, where the pattern and mechanism of the formation and breaking of CB involving Mg${}^{2+}$ are clearly seen. Interestingly, cofactor Mg${}^{2+}$ exhibits a unique role in the conformational change of KRAS-G12D, while having little influence on KRAS-WT and KRAS-G12C. As shown in Figure 1, for the KRAS-G12D case, Mg${}^{2+}$ can form CB with SER17 (oxygen labeled ‘O2’, see Figure A2), ASP57 (O1, O2) of KRAS-G12D and oxygens O1B and O2B of GDP (state 1). During the first 500 ns, $CB_{Mg^{2+}-SER17\left(O2\right)}$ is in a dynamic fluctuation and can be formed and broken (state 2). In the next 1.5 μs, bonds $CB_{Mg^{2+}-GDP\left(O2B\right)}$ and $CB_{Mg^{2+}-ASP57\left(O2\right)}$ are broken. Finally, Mg${}^{2+}$ forms strong CB with GDP (O1B), ASP57 (O1) and SER17 (O2) (state 3). In KRAS-WT and KRAS-G12C cases, the interaction patterns of Mg${}^{2+}$ with KRAS and GDP are similar to each other, but they are very different from the KRAS-G12D case. In particular, Mg${}^{2+}$ forms CB with SER17 (O2) and O2A, O1B, and O2B atoms of GDP, while the lifetimes of Mg${}^{2+}$-SER17 (O2) and Mg${}^{2+}$-GDP (O1B) are rather short. Once the $CB_{Mg^{2+}-SER17\left(O2\right)}$ bond was broken, Mg${}^{2+}$ was subsequently released from the binding site of KRAS-SER17.

### 2.4. GDP Plays an Important Regulatory Role in the Conformational Change of SW-I

**Figure A8:**
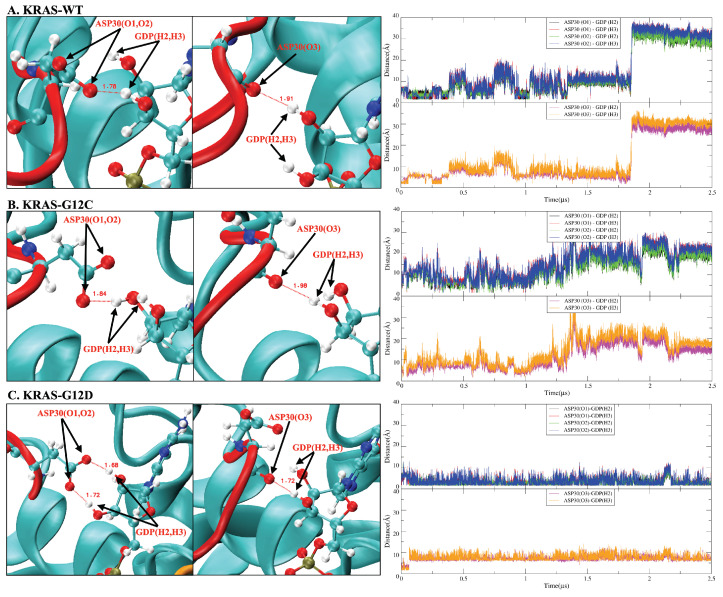
The hydrogen bond interaction between ASP30 of KRAS and GDP. (**A**) KRAS-WT case. Left side: representative snapshot of $HB_{ASP30(O1/O2)-GDP(H2/H3)}$ and $HB_{ASP30(O3)-GDP(H2/H3)}$, right side: time evolution of atom–atom distance d(t) corresponding to the left side; (**B**) KRAS-G12C case. Left side: representative snapshot of $HB_{ASP30(O1/O2)-GDP(H2/H3)}$ and $HB_{ASP30(O3)-GDP(H2/H3)}$, right side: time evolution of atom-atom distance d(t) corresponding to the left side; (**C**) KRAS-G12D case. Left side: representative snapshot of $HB_{ASP30(O1/O2)-GDP(H2/H3)}$ and $HB_{ASP30(O3)-GDP(H2/H3)}$, right side: time evolution of atom–atom distance d(t) corresponding to the left side.

In addition to the aforementioned CB/HB interactions in KRAS-GDP-Mg${}^{2+}$ complex, the HB interactions were also investigated between SW-I and GDP (Figure A8). We found that the HB interaction of SW-I (ASP30) with GDP is important for the transition of the SW-I conformation. The H2 and H3 atoms of GDP are very sensitive: when they form hydrogen bonds with the oxygen atoms of ASP30, these HBs between ASP30 and GDP can limit the conformational change of SW-I within a small range. From the time evolution of selected atom–atom distances d(t) in Figure A8, we can see that the strength of the HB interaction between ASP30 and GDP are KRAS-G12D > KRAS-WT > KRAS-G12C. This matches the RMSD variation of each KRAS in Figure A3A. Correspondingly, KRAS-G12D exhibits extreme conformational stability during intervals of at least 2.5 μs of the simulations. We also observed SW-I of KRAS-WT opening largely, while the HB interaction between ASP30 and GDP breaks around 1.9 μs. Finally, since ASP30 has the weakest HB interaction with GDP in the KRAS-G12C case, the latter exhibits large conformational changes around 1 μs.

### 2.5. The Dominant Conformations of Wild-Type and Mutated KRAS

**Figure 2:**
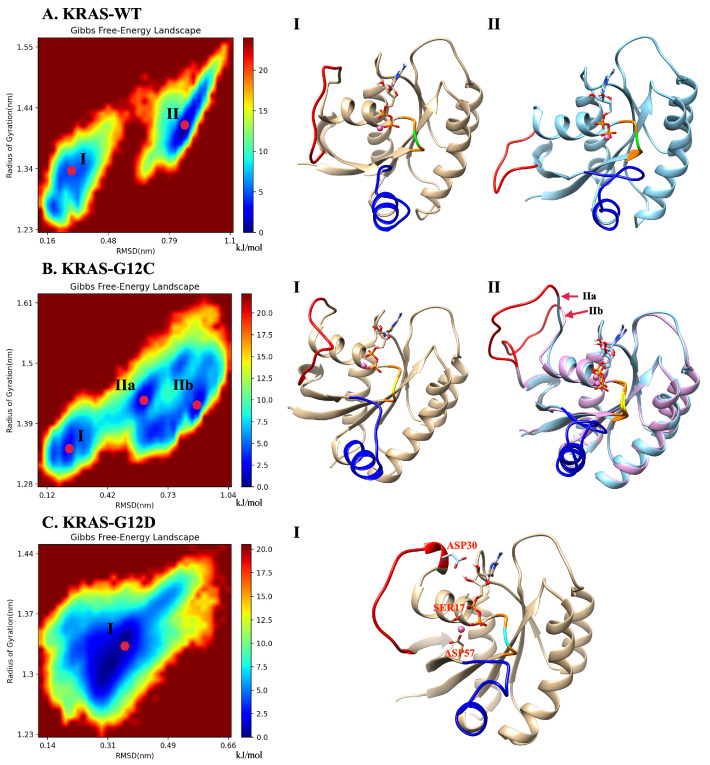
Gibbs free energy landscapes and representative conformations: (**A**) landscapes of the KRAS-WT-GDP-Mg${}^{2+}$ complex and the representative dominant conformations of basins I and II; (**B**) landscapes of KRAS-G12C-GDP-Mg${}^{2+}$ complex and the representative dominant conformations of basins I and II; (**C**) landscapes of the KRAS-G12D-GDP-Mg${}^{2+}$ complex and the single representative dominant conformation (I). Selected groups: backbone atoms of residue 10 to 72; GDP and the Mg${}^{2+}$ binding on KRAS. Red: SW-I; Blue: SW-II; Orange: P-loop; Green: GLY12; Yellow: CYS12; Cyan: ASP12.

**Figure A9:**
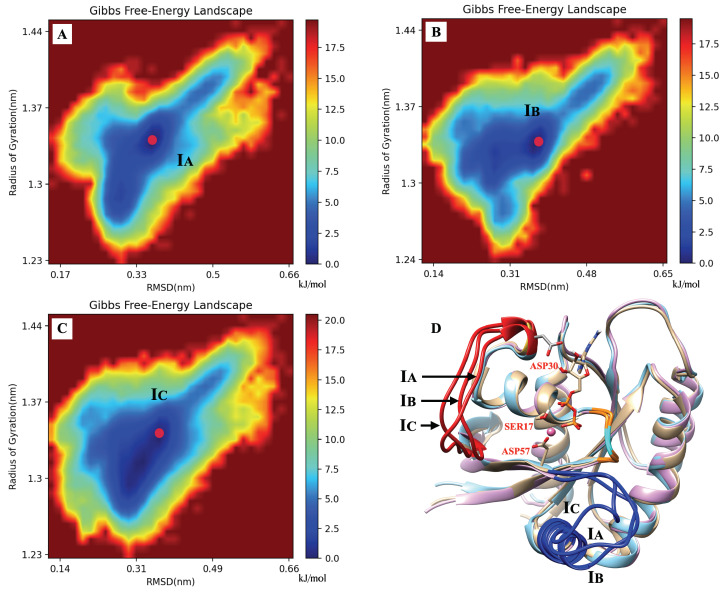
The system including KRAS-G12D reveals that the two generated trajectories of 2.5 μs produce a very similar free-energy surface (cases **A**,**B**) with the single stable state located approximately at the same coordinates. The average (**C**) is reported in Figure 3 of the manuscript. Stable states indicated by IA, IB and IC. Snapshot (**D**) reveals small differences for the three configurations corresponding to the stable state. Note: here, we choose SW-I, SW-II, GDP and Mg${}^{2+}$ to calculate RMSD and $R_{g}$, so the RMSD value here is larger than in Figure A3A. In Figure A3A, the RMSD is calculated for the full KRAS protein.

Gibbs free energy profiles are of high significance to characterize the dominant conformations of KRAS during simulation. In the present work, such analytical tools will allow us to directly track the effects of GLY12 mutations on the dominant conformations of the KRAS. Since the main movement of the protein is concentrated in SW-I and SW-II and our main research object is KRAS-GDP-Mg${}^{2+}$ ternary complex, we chose to compute Gibbs free energy landscapes by using two specific variables, including RMSD and radius of gyration, see below. The method employed to obtain the free energy profiles is the so-called “principal component analysis” [61,62], where the components RMSD and $R_{g}$ work as reaction coordinates (Figure 2). In the KRAS-WT case, we detected two free energy basins (I, II). Basin II is the one with lowest free energy (set to 0 kJ/mol), and it was chosen as the reference so that basin I shows a barrier of 4.6 kJ/mol, when transitions from basins I to II are considered. These two Gibbs free energy basins represent the two main stable states of KRAS-WT during MD simulations. We can obtain from the MD trajectories that in state I, SW-I is slightly open, while in the dominant basin II, SW-I opens largely and SW-II tends to be closer to the P-loop (region containing the mutated codon 12 and depicted in orange color). Several Gibbs free energy basins were also detected in the KRAS-G12C. In this case, the representative conformation of basin I is similar to the corresponding one in KRAS-WT, while basin II is divided into several small energy basins, with IIa and IIb as examples. The dominant conformation is now located on basin IIb (0 kJ/mol), with basin I at 2.7 kJ/mol and basin IIa at 1.0 kJ/mol. These basins are separated by low free energy barriers and they are moderately easy to access by the system. The differences between basins IIa and IIb are mainly on SW-I, with the SW-I conformation of IIb being more open than that of IIa. The comparison between KRAS-WT, KRAS-G12C and KRAS-G12D shows a clear difference: a single Gibbs free energy basin is found for KRAS-G12D, which indicates that such species have only one dominant stable conformation. As it can be seen from the representative crystal structure of this particular free energy basin I (Figure 2C), this dominant conformation of KRAS-G12D corresponds to SW-I being slightly open, while the distance between SW-II and P-loop is relatively tight. This is because the strong CB interaction of Mg${}^{2+}$ with GDP (O1B), SER17 (O2) and ASP57 (O1) can stabilize the distance between SW-II, GDP and the α-helix of KRAS, where SER17 is located. We also observed that the ASP12 shows strong interaction with GLY60 and GLN61, both located at SW-II. At the same time, hydrogen atoms H2 and H3 of GDP can form stable HB interactions with ASP30, helping to stabilize the distance between SW-I and GDP. To corroborate the full convergence of the free energy landscapes reported in Figure 2, we report in Appendix A (Figure A9) the contribution of the two independent 2.5 μs trajectories that we employed in this work (A,B), compared with their average (C), as shown in Figure 2C.

### 2.6. The Two Unique Druggable Dynamic Pockets on KRAS-G12D

**Figure 3:**
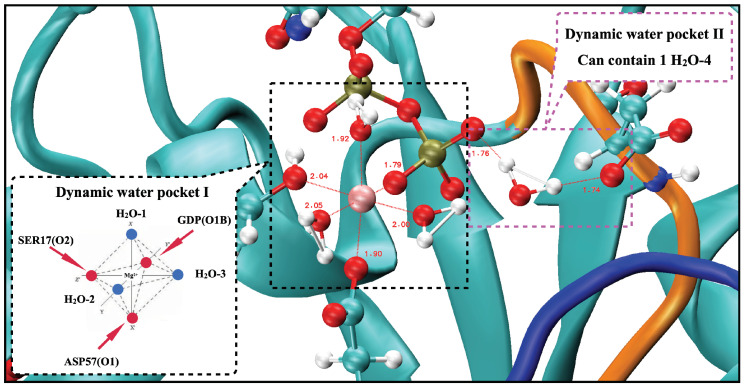
Snapshot of two dynamic pockets on the surface of KRAS-G12D-GDP-Mg${}^{2+}$ ternary complex.

**Figure A10:**
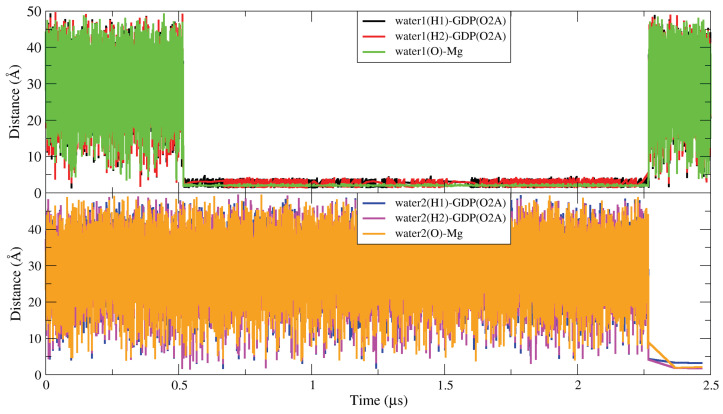
The dynamic change law of water molecules in the dynamic water pocket I.

**Figure A11:**
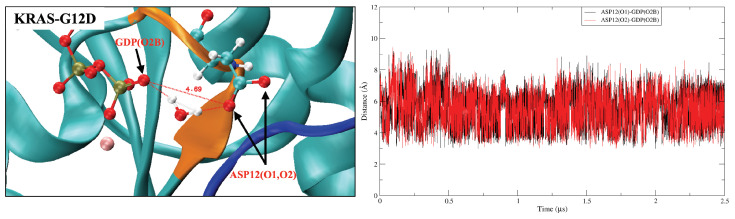
The dynamic water pocket II.

**Figure A12:**
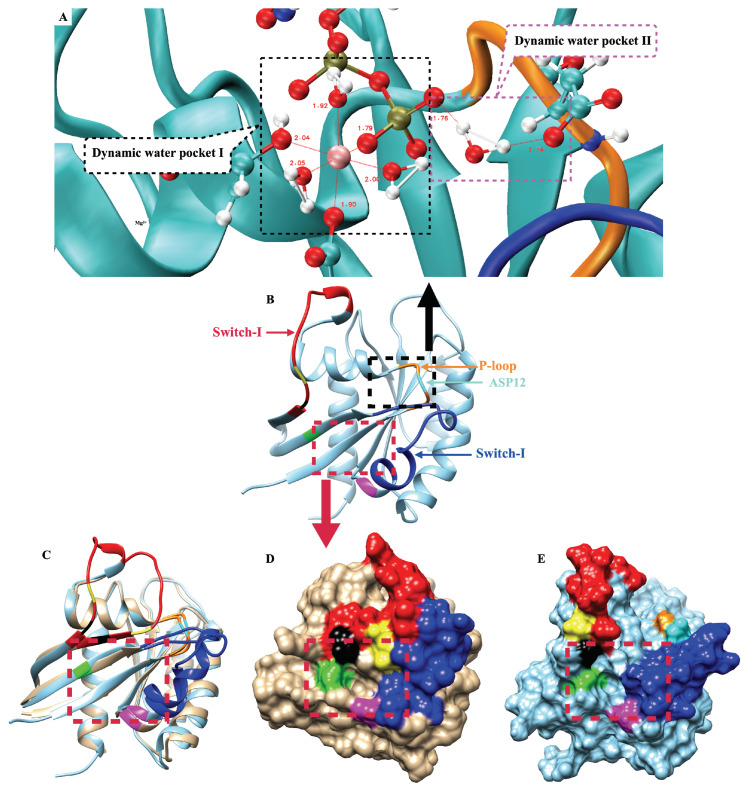
(**A**) The dynamic pockets I and II. (**B**) The most dominant conformation in our simulation results, black box: dynamic pockets I and II; red box: the pocket III. (**C**) Comparison of our simulation results with the crystal structure 6GJ7 (BI-2852, GDP, water and Mg2+ were hidden; red box: the location of pocket III, PDB ID code 6GJ7). (**D**) The surface version of crystal structure 6GJ7 (BI-2852 was hidden, PDB ID code 6GJ7). (**E**) The surface version of the most dominant conformation in our simulation results. Yellow: GLU37, Black: SER39, Green: ASP54, Magenta: THR74. Red box: the targetable pockets reported by Kessler et al. [28] and in the present KRAS-G12D simulation case.

From the previous analysis, KRAS-G12D exhibited a stable conformation that was significantly different from those of KRAS-WT and KRAS-G12C, and Mg${}^{2+}$ exhibited a unique binding mode in the KRAS-G12D surface. Mg${}^{2+}$ can form strong CB interactions with GDP (O1B), SER17 (O2) and ASP57 (O1). Well-established experimental results [51,52] showed that the octahedral structure is the most stable structure for Mg${}^{2+}$ first shell coordination, which is fully consistent with our simulation results. When we take into account the interaction of KRAS-GDP-Mg${}^{2+}$ complexes in aqueous Mg-Cl solution, we find the dynamic water pocket I in the Mg${}^{2+}$ binding area (left side of Figure 3). We call it “dynamic” in the following sense: in the octahedral structure of Mg${}^{2+}$, the fluctuation frequencies of the coordinated water molecules (in positions H${}_{2}$O-1, H${}_{2}$O-2 and H${}_{2}$O-3) are lower than those of free water molecules in solution, i.e., the former remain in their positions for much longer periods of time than the latter (nanoseconds compared to the picosecond time scale for bulk water HB dynamics [63]). At the same time, the coordinated water molecules can be exchanged with other water molecules in the solution (Figure A10). Further analysis of the trajectory uncovered another dynamic water pocket II, which is located between the phosphate group of GDP and the ASP12 (right side of Figure 3). This dynamic pocket can accommodate only one H${}_{2}$O molecule, through HB interactions of H${}_{2}$O with GDP (O2B) and ASP12 (O1/O2) (Figure A11). Further, the water molecule in this pocket is in a dynamic fast exchange with other water molecules in the solution. For the sake of corroboration of the newly observed pockets I and II, we included in Appendix A (see Figure A12) a comparison of the location of the pocket reported by Kessler et al. [28] with the pockets reported in the present work.

### 2.7. Structure-Based Drug Design and Targeting the Dynamic Water Pockets on KRAS-G12D

**Figure 4:**
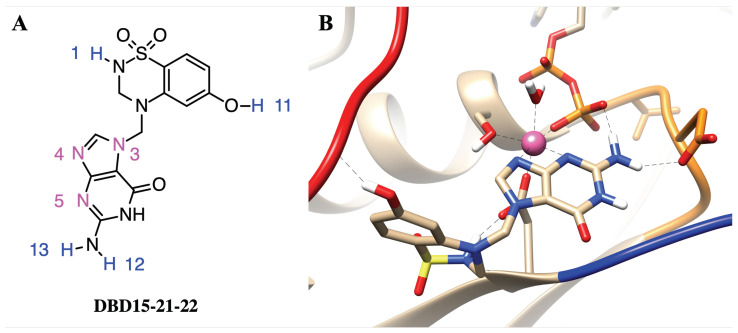
(**A**) Chemical structure of designed inhibitor DBD15-21-22 and (**B**) sketch of the interaction of details DBD15-21-22 with KRAS-G12D. Colors as in Figure 2.

In order to target the newly revealed binding pockets, we designed a drug in silico able to block GDP at its main cavity in a permanent way. There are several identified ways to target KRAS, the following being the most relevant [64]: inhibition of RAS expression, interference with RAS post-translational modifications, inhibition of RAS function or targeting specific downstream effectors. Furthermore, our strategy is different from that of the designers of recent drug *Sotorasib* [65]. We observed that in recent years, selective binding ligands for Mg${}^{2+}$ have gradually become known [66]. In dynamic water pocket I, H${}_{2}$O-2 and H${}_{2}$O-3 molecules can exchange with other water molecules in the aqueous solution. The two oxygen atoms of H${}_{2}$O-2 and H${}_{2}$O-3 are on the same horizontal plane, and the distance between the two oxygen atoms is comparable to the size of the bidentate ligand. This feature makes it possible to design the selective inhibitors that can replace these two water molecules and target this dynamic water pocket I. Herein, we designed a series of inhibitors for these two dynamic water pockets using a benzothiadiazine, specifically 3,4-dihydro-1,2,4-benzothiadiazine-1,1-dioxide (C_7_H_8_N_2_O_2_S, DBD), as a template. The most suitable species obtained is designed as DBD15-21-22 (see Figure 4A) since it is a DBD derivative, similar to some of those reported in Ref. [67].

### 2.8. DBD15-21-22 Can Target KRAS-G12D and Bind Dynamic Water Pockets I and II

**Figure A13:**
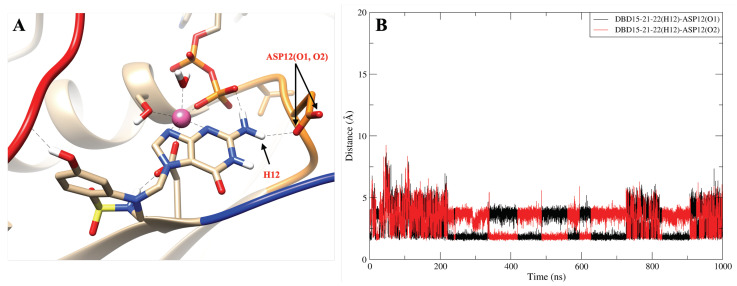
The hydrogen bond interaction between DBD15-21-22 (H12) and ASP12 (O1, O2). (**A**) Representative snapshot of $HB_{DBD15-21-22(H12)-ASP12(O1/O2)}$; (**B**) distance between DBD15-21-22 (H12) and ASP12 (O1, O2).

**Figure A14:**
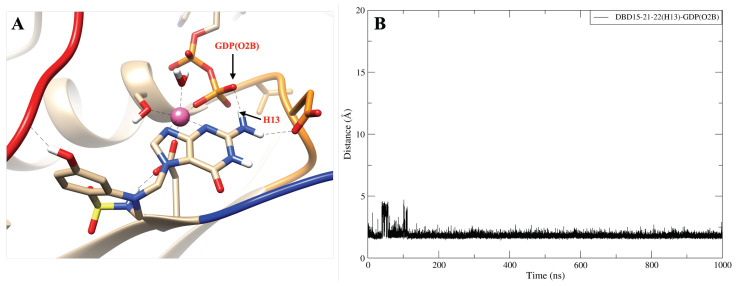
The hydrogen bond interaction between DBD15-21-22 (H13) and GDP (O2B). (**A**) Representative snapshot of $HB_{DBD15-21-22(H13)-GDP(O2B)}$; (**B**) distance between DBD15-21-22 (H13) and GDP (O2B).

**Figure A15:**
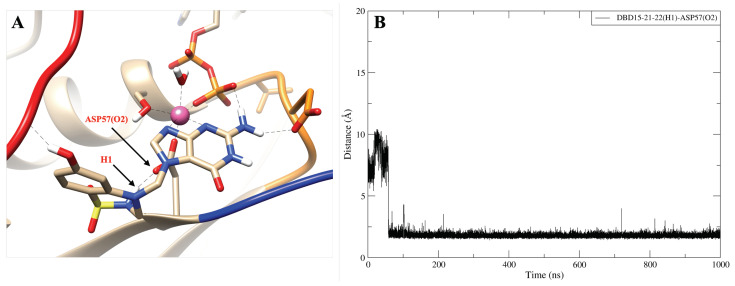
The hydrogen bond interaction between DBD15-21-22 (H1) and ASP57 (O2). (**A**) Representative snapshot of $HB_{DBD15-21-22(H1)-ASP57(O2)}$; (**B**) distance between DBD15-21-22 (H1) and ASP57 (O2).

**Figure A16:**
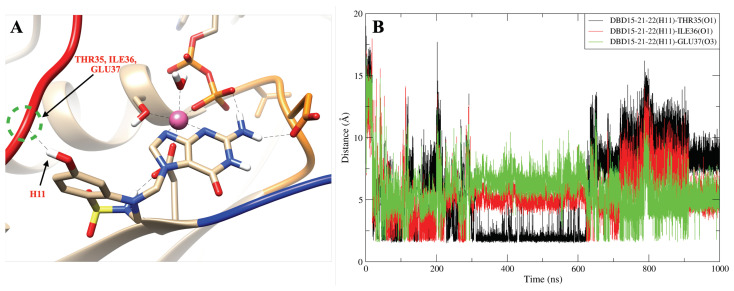
The DBD15-21-22 (H11) can form hydrogen bond interactions with THR35, ILE36 and GLU37. (**A**) Representative snapshot of DBD15-21-22 (H11) forming hydrogen bond interactions with THR35, ILE36 and GLU37; (**B**) distance between DBD15-21-22 (H11) and three SW-I residues (THR35, ILE36, GLU37).

**Figure A17:**
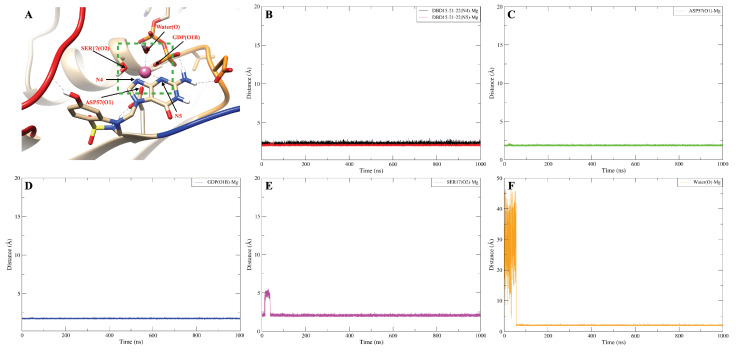
DBD15-21-22 binding on the dynamic water pocket I and stabilized the octahedral structure of Mg${}^{2+}$. (**A**) Representative snapshot of DBD15-21-22 (N4,N5) form coordination bond with Mg${}^{2+}$; (**B**) distance between DBD15-21-22 (N4,N5) and Mg${}^{2+}$; (**C**) distance between ASP57 (O1) and Mg${}^{2+}$; (**D**) distance between GDP (O1B) and Mg${}^{2+}$; (**E**) distance between SER17(O2) and Mg${}^{2+}$; (**F**) distance between water molecular in position H${}_{2}$O-1 and Mg${}^{2+}$.

**Figure A18:**
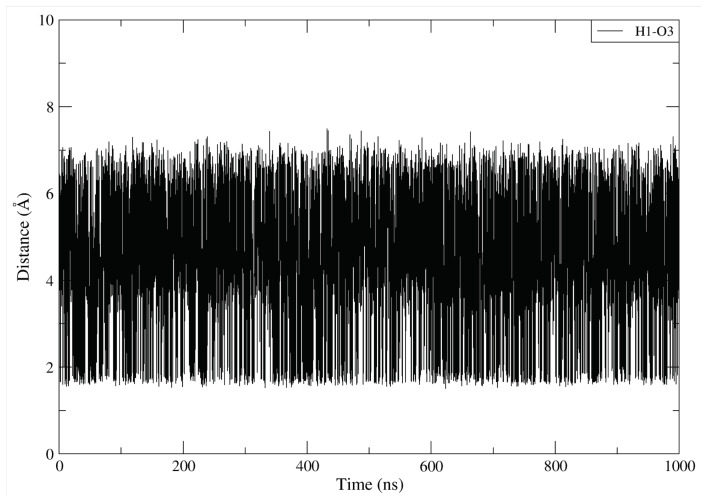
The distance between H1 and O3 atoms in DBD15-21-22.

To further study the structure of the DBD15-21-22 interaction with KRAS-G12D, we performed microsecond time scale MD simulations of DBD15-21-22 together with KRAS-G12D. As we can see from the MD results, the DBD15-21-22 is tightly bound to its binding pocket (Figure 4B). The N4 and N5 of DBD15-21-22 can replace H${}_{2}$O-2 and H${}_{2}$O-3 in the octahedral structure of Mg${}^{2+}$ and then specifically bind to the dynamic water pocket I. Benefiting from the location and size of the amino group ($-NH_{2}$) near the N4 and N5, the $-NH_{2}$ group can perfectly replace the H${}_{2}$O-4 in the dynamic water pocket II. These two concerted actions enable DBD15-21-22 to bind tightly and specifically to the two dynamic water pockets on the KRAS-G12D-GDP-Mg${}^{2+}$ complex. In addition, other active sites in DBD15-21-22 can also form stable HB interactions with other residues of KRAS-G12D. In particular (see Figure 4A), the H1 atom of DBD15-21-22 can form HB with ASP57 (O2) and the H11 atom on the phenolic hydroxyl (−OH) group can form HB with multiple residues on SW-I to lock the conformational change of SW-I. In order to estimate the detailed HB and CB interactions between DBD15-21-22 and its unique druggable pocket on KRAS-G12D, we display the time evolution of selected atom–atom distances d(t) in Figure A12, Figure A13, Figure A14, Figure A15, Figure A16, Figure A17 and Figure A18.

### 2.9. DBD15-21-22 Is Harmless to KRAS-WT

**Figure 5:**
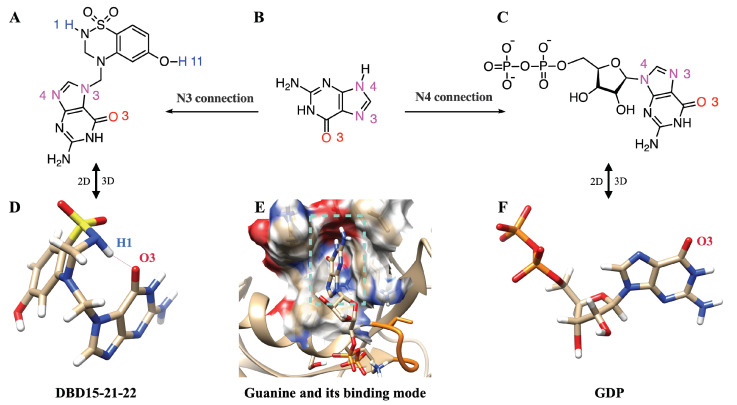
DBD15-21-22 cannot bind to the GDP/GTP binding pocket of KRAS-WT: (**A**) 2D structure of DBD15-21-22; (**B**) 2D structure of guanine; (**C**) 2D structure of GDP; (**D**) 3D structure of DBD15-21-22 in water; (**E**) GDP-binding model of KRAS-WT; (**F**) 3D structure of GDP in water.

As we can see in Figure 5A, in the structure of DBD15-21-22 there exists a guanylate moiety but the binding patterns are quite different from GDP/GTP. When GDP/GTP binds to the GDP/GTP binding pocket (Figure 5E), the O3 of guanine goes deep inside the pocket. DBD15-21-22 also has a guanine group, which is similar to GDP/GTP. The difference is that in DBD15-21-22 (Figure 5A), the DBD part is connected to the N3 atom of guanine; in contrast, the β-D-ribofuranosyl in the GDP structure is connected to the N4 atom of guanine (Figure 5C). The drug we designed takes advantage of this subtle difference, and prevents the guanine structure in the DBD15-21-22 from binding to the GDP/GTP pocket through the steric hindrance of the DBD part in the DBD15-21-22. Moreover, we investigated the three-dimensional structure of DBD15-21-22 and GDP in an aqueous solution (Figure 5D,F). DBD15-21-22 can form intramolecular hydrogen bonds (Figure 5D), further preventing DBD15-21-22 from binding to the binding site of GDP/GTP on KRAS, and thereby minimizing the toxicity to KRAS-WT.

**Figure A19:**
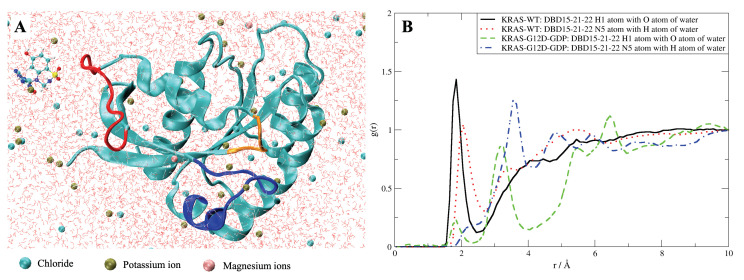
(**A**) Snapshot of DBD15-21-22 simulation with GDP/GTP free KRAS-WT. (**B**) RDF of DBD15-21-22 with water.

To further verify the low toxicity of DBD15-21-22 to KRAS-WT, we also performed MD simulations of DBD15-21-22 together with GDP free KRAS-WT. From the simulation results, we can see that DBD15-21-22 was unable to bind to the GDP/GTP binding pocket on KRAS-WT but was dissolved in an aqueous solution most of the time (Figure A19A).This finding is further verified by the radial distribution function (RDF) of the DBD15-21-22 interaction with aqueous solutions (Figure A19B). We can observe that DBD15-21-22 dissolves well in water, with hydrogen H1 and nitrogen N5 of DBD15-21-22 both forming HBs with H${}_{2}$O. However, in the KRAS-G12D-DBD15-21-22 simulation case, the HB interaction of DBD15-21-22 with water was interrupted such that DBD15-21-22 was well bound to the druggable pocket on the surface of KRAS-G12D, as described in Figure 4B).

## 3. Methods and Materials

Our main computational tool has been microsecond scale molecular dynamics. In MD, after the choice of reliable force fields, the corresponding Newton’s equations of motion are integrated numerically [68], allowing us to monitor each individual atom in the system in a wide variety of setups, including liquids at interfaces in solid walls or biological membranes, among others [69,70]. We fixed the number of particles, the pressure and the temperature of the system, while the volume was adjusted accordingly. MD can model hydrogens at the classical [71,72] or quantum levels [73] and, in addition to energetic and structural properties, MD provides access to time-dependent quantities, such as the diffusion coefficients, rotational and vibrational motions [74] or spectral densities [75], enhancing its applicability. In the present work, we conducted MD simulations of three KRAS isoforms with sequences represented in Figure A1. Each system contains one isoform of KRAS-GDP complex fully solvated by 5697 TIP3P water molecules [76] in potassium chloride solution at the human body concentration (0.15 M) and magnesium chloride solution concentration (0.03 M), yielding a system size of 19,900 atoms. All MD inputs were generated using the CHARMM-GUI solution builder [77,78,79], and the CHARMM36m force field [80] was adopted for KRAS-GDP-Mg${}^{2+}$ interactions. The force field used also includes the parameterization of the species GDP (it can be searched as “GDP” in the corresponding CHARMM36m topology file: https://www.charmm-gui.org/?doc=archive&lib=csml (accessed on 10 October 2021)). All bonds involving hydrogens were set to fixed lengths, allowing fluctuations of bond distances and angles for the remaining atoms. The crystal structure of GDP-bound KRAS proteins was downloaded from the RCSB PDB Protein Data Bank [81], file name “4obe”. The three sets of KRAS-GDP complex (wild type, G12C mutate and G12D mutate) were solvated in a water box, all systems were energy minimized for 50,000 steps and well equilibrated (NVT ensemble) for 250 ps before generating the production MD. Two independent production runs were performed within the NPT ensemble for 2.5 μs. All meaningful properties were either averaged from the two runs.The pressure and temperature were set at 1 atm and 310.15 K respectively, in order to simulate the human environment. In all MD simulations, the GROMACS/2021 package was employed [82]. Time steps of 2 fs were used in all production simulations, and the particle mesh Ewald method with a Coulomb radius of 1.2 nm was employed to compute long-ranged electrostatic interactions. The cutoff for Lennard–Jones interactions was set to 1.2 nm. Pressure was controlled by a Parrinello–Rahman piston with damping coefficient of 5 ps${}^{-1}$, whereas temperature was controlled by a Nosé–Hoover thermostat with a damping coefficient of 1 ps${}^{-1}$. Periodic boundary conditions in three directions of space were taken. We employed the “gmx-sham” tool of the GROMACS/2021 package to perform the Gibbs free energy landscape analysis. Other alternatives, such as transition path sampling [83,84,85] or metadynamics [86,87], were not considered here because of their high computational cost for large systems. The designed inhibitor DBD15-21-22 was parameterized using CgenFF [88,89] to obtain the CHARMM-compatible topology and parameter files. Further, we switched to run another four 1 μs of MD simulations to study the interaction of the inhibitor DBD15-21-22 with KRAS-G12D and to verify that DBD-15-21-22 is harmless on the wild-type KRAS. These four new MD simulations have the same setup as the previous ones. Moreover, the software VMD [90] and UCSF Chimera [91] were used for trajectory analysis and visualization.

As key computed quantities in this work, the radius of gyration ($R_{g}$) is defined in Equation (1):

(1) $$R_{g}=\sqrt{\frac{\sum_{i}{∥r_{i}∥}^{2}m_{i}}{\sum_{i}m_{i}}},$$

where $m_{i}$ is the mass of atom *i*, and $r_{i}$ the position of the same atom with respect to the center of mass of the selected group. Root mean square deviations (*RMSD*) are defined by Equation (2):

(2) $$RMSD\left(t\right)\equiv \sqrt{\frac{1}{N}\sum_{i=1}^{N}\delta_{i}^{2}\left(t\right)},$$

where $\delta_{i}$ is the difference in distance between the atom *i* (located at $x_{i}\left(t\right)$) of the catalytic domain and the equivalent location in the crystal structure, and finally root mean square fluctuations (*RMSF*) are defined by Equation (3):

(3) $$RMSF_{i}\equiv \sqrt{\frac{1}{\Delta t}\sum_{t_{j}=1}^{\Delta t}{\left(x_{i}\left(t_{j}\right)-{\tilde{x}}_{i}\right)}^{2}},$$

where ${\tilde{x}}_{i}$ is the time average of $x_{i}$, and Δt is the time interval where the average is taken.

## 4. Conclusions

**Figure 6:**
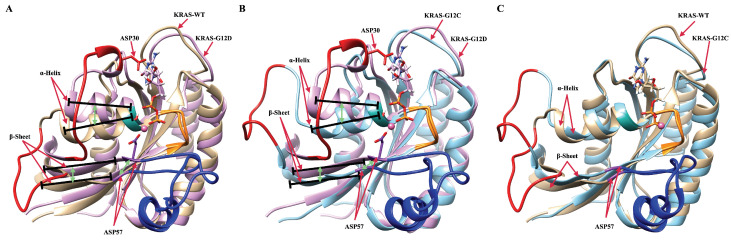
Alignment of three representative dominant structures of the GDP-bound KRAS-WT, KRAS-G12C and KRAS-G12D. (**A**) Alignment of GDP-bound KRAS-WT and GDP-bound KRAS-G12D; (**B**) alignment of GDP-bound KRAS-G12C and GDP-bound KRAS-G12D; (**C**) alignment of GDP-bound KRAS-WT and GDP-bound KRAS-G12C.

To summarize the main findings of the work, let us remark that KRAS mutations are widely present in many cancer cases, but there exist usually only molecular-scale differences between different KRAS mutants, such as in the one site GLY12 mutation. This means the direct information on atomic interactions and local structures at the all-atom level of KRAS can be extremely useful for oncogenic KRAS research. Based on powerful computer simulation tools, we focused our analysis on the local structure of KRAS proteins when associated with Mg${}^{2+}$, GDP, and water. After systematic analysis of meaningful equilibrated data, we can reveal the dynamic differences between KRAS-WT and the mutated species KRAS-G12C and KRAS-G12D at the all-atom level. There exist several important intermolecular interactions affecting the behavior of KRAS. The most relevant features are (1) the strong coordination interactions between Mg${}^{2+}$, SER17, ASP57 and GDP in KRAS-G12D; (2) the mutation of GLY12 enhances the interaction between P-loop and SW-II domain, with the enhancement magnitude ordered as KRAS-G12D > KRAS-G12C; and (3) the GLY12 mutation also affects the interaction between GDP and SW-I. Although GLY12 mutation can enhance the interaction of the P-loop with SW-II, in the KRAS-G12C case, the interaction of GDP with SW-I was almost totally eliminated, whereas the G12D mutation greatly enhanced the interaction of GDP with SW-I. In Figure 6, we show the alignment of three representative dominant structures of the GDP-bound KRAS-WT, KRAS-G12C and KRAS-G12D. As shown in Figure 6A, the downward sliding of the α-helix and β-sheet structures of KRAS-WT promoted the large opening of the SW-I domain. When GLY12 was mutated to ASP12, the interaction between P-loop and SW-II was largely enhanced, then the relative position of P-loop and SW-II was shortened and stabilized. This promotes strong coordination interactions between Mg${}^{2+}$, SER17, ASP57 and GDP, which further stabilizes the relative positions of β-Sheet and SW-II in the protein structure. Also benefiting from the hydrogen bond interaction between ASP30 and GDP, the fluctuation of SW-I was limited to a small range, which led to a slightly open stable conformation of SW-I in KRAS-G12D. When GLY12 was mutated to CYS12, although the interaction between P-loop and SW-II was enhanced, it was not enough to stabilize the relative positions of α-Helix, β-Sheet and SW-II in the structure of KRAS, and the conformational changes were similar to those of the KRAS-WT.

After deciphering the laws of conformational changes of different KRAS mutants, two druggable dynamic water pockets on KRAS-G12D-GDP-Mg${}^{2+}$ ternary complex were revealed. We designed a specific inhibitor DBD15-21-22 for these dynamic water pockets, with the aim to lock the KRAS-G12D in the “off” (inactive) state, i.e., when the protein is unable to perform signaling processes and eventually develops cancer. Benefiting from the fact that H1 and O3 atoms of DBD15-21-22 can form intramolecular hydrogen bonds in aqueous solution, we detected no binding effects of DBD15-21-22 toward the GDP/GTP binding pocket of KRAS-WT, whereas our molecular dynamics simulation results show that this KRAS-G12D inhibitor can efficiently bind to the targeting pocket located between SW-I, SW-II and P-loop, thereby efficiently locking KRAS-G12D in the “off” state. Overall, our work provides a new druggable pocket and a reliable protocol for the development of specific inhibitors targeting KRAS-G12D.

## Data Availability

Not applicable.

## Appendix A

### Appendix A.1. Initial Setups and Data for Molecular Dynamics Simulations

The three classes of KRAS proteins that were considered in this work are reported in Figure A1, where the initial structure of KRAS-WT, was acquired from PDB bank (4obe.pdb). We also show the octahedral structure of Mg${}^{2+}$ in the crystal structure, consistent with relevant experimental studies [51,52]. In order to make clear the atomic sites that will be described and analyzed in the article, we represent sketches of GDP and the main amino acid structures analyzed in the present work in Figure A2.

In Figure A3, and from the RMSD values as a function of time, we can see that the conformation of KRAS-G12D is stable during the whole simulation process (total statistics 5 μs). The difference from KRAS-G12D is that the conformation of KRAS-WT protein changes greatly in about 1.9 μs, and the conformation of KRAS-G12C protein changes greatly in about 1 μs. The RMSF results show the flexibility of amino acid residues in the KRAS structure. From the RMSF results, it can be seen that the structurally active regions of KRAS protein are mainly Switch-I (SW-I) and Switch-II (SW-II) domains. Notably, the flexibility of amino acid residues in the SW-I domain of KRAS-G12D is around twofold smaller than that of KRAS-WT and KRAS-G12C.

### Appendix A.2. Atomic Distances and KRAS Conformations

Throughout the work, it is very important to make sure that binding connections between specific atomic sites are stable enough to be considered as meaningful interactions so that monitoring the instantaneous distances between tagged species is of crucial importance. In our case, the computation of radial distribution functions is not suitable in most cases, given the specificity of the atomic sites involved in the relevant bindings between ions, amino acids and drugs. The series of distances are reported in Figure A4, Figure A5, Figure A6, Figure A7 and Figure A8, Figure A10, Figure A11 and Figure A13, Figure A14, Figure A15, Figure A16, Figure A17 and Figure A18, together with snapshots corresponding to the configurations of highest biophysical relevance. We mainly monitor hydrogens bonds and coordination bonds.

For instance, in Figure A6, we compare the effect of different mutations on the interactions between amino acid residues (VAL8-THR58) in the KRAS protein. In KRAS-WT, the HB interaction can last ∼2 μs. When the GLY12 mutated to CYS, the HB interaction between VAL8 and THR58 have been enhanced, interestingly, VAL8 (O3) prefers to form hydrogen bonds with THR58 (H2). In KRAS-G12D case, the hydrogen bond interaction between VAL8 and THR58 is significantly enhanced compared to KRAS-WT and KRAS-G12C. In Figure A7, the coordination bond analysis of Mg${}^{2+}$ is shown. In KRAS-WT case, Mg${}^{2+}$ cannot form a CB with ASP57; in the first ∼300 ns, Mg${}^{2+}$ can form CB with SER17 (O2) and GDP (O2A,O1B,O2B) and the $CB_{Mg^{2+}-SER17\left(O2\right)}$ and $CB_{Mg^{2+}-GDP\left(O1B\right)}$ are broken, while the $CB_{Mg^{2+}-GDP\left(O2A\right)}$ and $CB_{Mg^{2+}-GDP\left(O2B\right)}$ are very stable. In KRAS-G12C case, the situation is similar to KRAS-WT. The only difference is that the coordination interaction between Mg${}^{2+}$ and SER17 (O2)/GDP (O1B) can last ∼400 ns. Finally, in the KRAS-G12D case, the difference are that Mg${}^{2+}$ can form stable CB interaction with ASP57, SER17 and GDP (O1B). In the first ∼250 ns, the coordination interactions between Mg${}^{2+}$, SER17, ASP57 and GDP fluctuated, and then Mg${}^{2+}$ formed stable coordination bonds with SER17 (O2), ASP57 (O1) and GDP (O1B). From the selected atom-atom distance data of Figure A8, we can see that the structure of KRAS-G12C is similar to that of KRAS-WT, with the hydrogen bond interaction between GDP and ASP30 being very weak. However, when GLY12 is mutated to ASP12, the interaction between GDP and ASP30 has been significantly enhanced.

The dynamic change law of three water molecules (in positions H${}_{2}$O-1, H${}_{2}$O-2 and H${}_{2}$O-3) in the dynamic water pocket I is similar, so in Figure A10 we take the dynamic change law of water molecules at position H${}_{2}$O-1 as an example. From the selected atom–atom distance data, we can see that the exchange period between water molecules in dynamic water pocket I and solution water molecules is ∼1.7 μs. The dynamic water pocket II is consists of the oxygen atom (O2B) of GDP and the oxygen atoms of ASP12 (O1,O2), as shown in Figure A11 where in left side we show the representative snapshot of dynamic water pocket II and in the right side the distance between ASP12 (O1,O2) and GDP (O2B). As shown in the left side of Figure A11, the dynamic water pocket II can accommodate one water molecule. And this water molecular has a high exchange frequency with the water molecule in the aqueous solution, with an exchange period of about ∼100 ps. Therefore, the time evolution of atom–atom distance d(t) corresponding to the water molecule and GDP/ASP12 is not calculated.

In addition to the dynamic pockets I and II detailed above, the targetable pockets reported by Kessler et al. [28] were also monitored in our KRAS-G12D case. We show in Figure A12 a comparison of the main findings of the present work compared to those of Kessler et al. The difference in the location and characteristics of the two pockets is made clear.

Interestingly, some HB can switch: in Figure A13, the hydrogen atoms (H12) of DBD15-21-22 can form stable hydrogen bonds interaction with the oxygen atoms (O1,O2) of ASP12. And the ASP12(O1, O2) alternately form stable hydrogen bonds with DBD15-21-22(H12) (see Figure A13B). Conversely, in Figure A14B, the hydrogen atoms (H13) of DBD15-21-22 can form stable hydrogen bonds interaction with the oxygen atoms (O2B) of GDP.

The dynamics of the designed drug DBD15-21-22 (H1) indicates (Figure A15B) that at the beginning of the trajectory it is very far from the ASP57(O2) but after 50 ns it becomes close to the ASP57 (O2) and capable to form stable hydrogen bonds interactions. Further, as shown in Figure A16B, DBD15-21-22 (H11) can form hydrogen bonds with the oxygen atoms (THR35 (O1), ILE36 (O1) and GLU37 (O3)) of three different SW-I residues. Interactions of DBD15-21-22 with Mg${}^{+}$ ions are revealed to be very stable (see Figure A17). Further, from the distance between H1 and O3 atoms in DBD15-21-22 (see Figure A18), we can find that DBD15-21-22 can form intramolecular hydrogen bonds $HB_{H1-O3}$ when DBD15-21-22 is in aqueous solution.

Finally, Figure A19, shows selected radial distribution functions of DBD15-21-22 when dissolved with the KRAS-WT species compared to the KRAS-G12D-GDP case: in the former WT case, the inhibitor DBD15-21-22 is solvated by water instead of remaining at the surface of KRAS.

### Appendix A.3. Convergence of the Simulations

The equilibration of our simulation systems is reached after a few tens of nanoseconds. Afterwards, two long independent trajectories of 2.5 μs each are produced. In order to show the convergence of our results, we report the calculation of the free-energy surface corresponding to the KRAS-G12D mutation case from the two runs (A, B) and its average, (C) as shown in Figure A9. We include a superposition (D) of the stable state configuration of KRAS-G12D for the three sets reported (A, B, C).

**Table A1:** Amino acid compounds of the the KRAS proteins. Abbreviations as used in the text and in several figures.

Full Name	Abbreviation
Aspartate	ASP
Cysteine	CYS
Glutamine	GLN
Glycine	GLY
Lysine	LYS
Serine	SER
Threonine	THR
Valine	VAL
Proline	PRO

**Table A2:** Estimated lifetimes (in ns) of HB and CB interactions (Section 2 of main text, corresponding to Figure A4, Figure A5, Figure A6, Figure A7 and Figure A8). Total simulation time of 5 μs (2.5 μs per trajectory). We indicate in “Bond” the two amino acids or ion involved in the HB or CB and the corresponding atoms (hydrogen, oxygen) in parentheses. Double labels indicate averaged values.

Bond (Ion/Amino Acid (Atom))	KRAS-WT	KRAS-G12C	KRAS-G12D
GLY12 (H3)-GLY60 (O3)	1	-	-
GLY12 (H3)-GLN61 (O1)	7	-	-
CYS12 (H1)-GLY60 (O3)	-	1	-
CYS12 (H1)-GLN61 (O1)	-	8	-
ASP12 (O1,O2)-GLY60 (H3)	-	-	342
ASP12 (O1,O2)-GLN61 (H1,H2)	-	-	26
ASP12 (O1,O2)-GLN61 (H3)	-	-	113
VAL8 (O3)-THR58 (H2)	219	1050	422
VAL8 (O3)-THR58 (H3)	186	5	1403
Mg-ASP57 (O1)	-	-	2496
Mg-ASP57 (O2)	-	-	249
Mg-SER17 (O2)	252	329	2129
Mg-GDP (O2A)	2500	2500	-
Mg-GDP (O1B)	284	345	2500
Mg-GDP (O2B)	2500	2500	210
ASP30 (O1,O2)-GDP (H2,H3)	65	2	712
ASP30 (O3)-GDP (H2,H3)	30	2	48

**Table A3:** Estimated lifetimes (in ns) of HB and CB interactions (Section 2 of main text, corresponding to Figure A11, Figure A12, Figure A13, Figure A14 and Figure A15). Total simulation time of 1 μs. We indicate in “Bond” the atomic site of the designed new drug DBD-15-21-22 and the amino acid or ion involved in the HB or CB and the corresponding atoms in parentheses. Double labels indicate averaged values.

Bond (Drug/Ion/Amino Acid (Atom))	KRAS-G12D
DBD15-21-22 (H12)-ASP12 (O1,O2)	389
DBD15-21-22 (H13)-GDP (O2B)	970
DBD15-21-22 (H1)-ASP57 (O2)	932
DBD15-21-22 (H11)-THR35 (O1)	330
DBD15-21-22 (H11)-ILE36 (O1)	53
DBD15-21-22 (H11)-GLU37 (O3)	32
DBD15-21-22 (N4)-Mg	750
DBD15-21-22 (N5)-Mg	1000
ASP57 (O1)-Mg	1000
SER17 (O2)-Mg	964
GDP (O1B)-Mg	1000
Water (O)-Mg	931
